# The role of intestinal flora in metabolic dysfunction-associated steatotic liver disease and treatment strategies

**DOI:** 10.3389/fmed.2024.1490929

**Published:** 2025-01-07

**Authors:** Li Jun Wang, Jian Guang Sun, Shu Cheng Chen, Yu Li Sun, Yang Zheng, Jian Chao Feng

**Affiliations:** ^1^Department of Traditional Chinese Medicine, Binzhou Medical University, Yantai, China; ^2^The First Clinical Medical College of Shandong University of Traditional Chinese Medicine, Jinan, China; ^3^School of Nursing, The Hong Kong Polytechnic University, Kowloon, Hong Kong SAR, China; ^4^Department of Hepatology, Affiliated Hospital of Shandong University of Traditional Chinese Medicine, Jinan, China; ^5^Department of Acupuncture and Moxibustion, Zibo Hospital, Zibo, China

**Keywords:** intestinal flora, metabolic dysfunction-associated steatotic liver disease, probiotics, metabolism, review

## Abstract

Metabolic dysfunction-associated steatotic liver disease (MASLD) is a common multi-factorial liver disease, and its incidence is gradually increasing worldwide. Many reports have revealed that intestinal flora plays a crucial role for the occurrence and development of MASLD, through mechanisms such as flora translocation, endogenous ethanol production, dysregulation of choline metabolism and bile acid, and endotoxemia. Here, we review the relationship between intestinal flora and MASLD, as well as interventions for MASLD, such as prebiotics, probiotics, synbiotics, and intestinal flora transplantation. Intervention strategies targeting the intestinal flora along with its metabolites may be new targets for preventing and treating MASLD.

## Graphical abstract

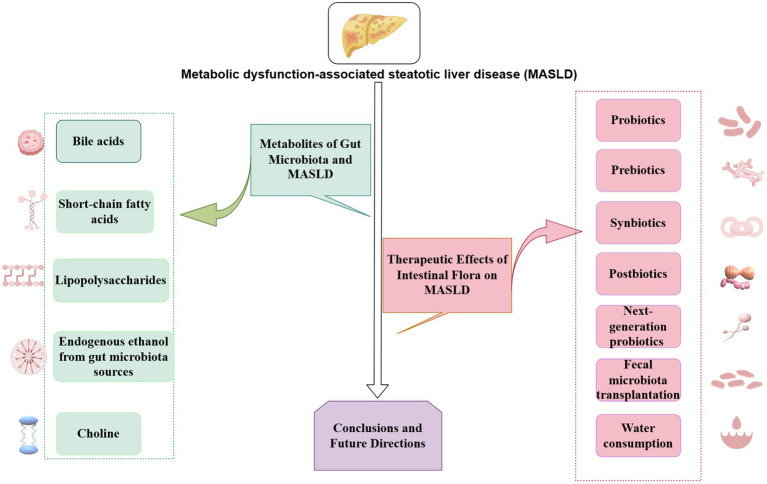

## 1 Introduction

Metabolic dysfunction-associated steatotic liver disease (MASLD), formerly known as non-alcoholic fatty liver disease, comprises a group of acquired metabolic stress-related liver diseases (1) and is characterized by the abnormal accumulation of liver fat and metabolic stress-induced liver damage, excluding viral infections, alcohol excessive use, and other factors. MASLD can gradually progress from isolated liver steatosis to metabolic dysfunction associated stem liver disease (MASH). If left untreated, MASH can further progress to cirrhosis, liver failure, and hepatocellular carcinoma (2). MASLD is related to metabolic syndromes such as insulin resistance, centripetal obesity, hypertension, and hyperlipidemia (3, 4). In addition, there is evidence to suggest that MASLD increases the risk of cardiovascular disease and chronic kidney disease (5, 6).

With changes in lifestyle, eating habits, and other factors, the incidence rate of MASLD has reached as high as 25%, and approximately two billion people worldwide are affected (7). MASLD is the most common chronic liver disease globally. The Middle East and South America show the highest incidence rates of 31.8 and 30.5%, respectively (8). The overall prevalence of MASLD in Asian countries is 29.6% and is increasing (9).

The pathogenesis of MASLD is unclear. In addition to fat accumulation, lipid oxidative stress, insulin resistance, gut microbiota, metabolites, and abnormal intestinal barrier function are also closely involved in the incidence and development of MASLD. The liver and the intestine have a close structural and functional relationship known as the “gut-liver axis.” Seventy five percent of the liver blood supply comes from the portal vein, making it the first organ to be exposed to the gut microbiota and metabolites via the portal blood supply (10). A normal intestinal barrier can prevent the transfer of gut microbiota, metabolites, or toxins outside the intestinal cavity. Dysregulation of the gut microbiota can affect its metabolites and intestinal permeability, causing gut microbiota translocation, overactivation of the immune system, and exacerbation of the occurrence and progression of MASLD. This article reviews the mechanisms of action and prevention methods of the gut microbiota and its metabolites in MASLD pathogenesis.

## 2 MASLD and changes in gut microbiota species

The gut microbiota is a multifaceted ecosystem that has a symbiotic relationship with the host, containing 1,000–1,500 species of approximately 10–10 trillion bacteria (10 times the number of human cells). Among these, Bacteroidetes and Firmicutes are dominant and are associated with steatosis (11). Clinical trials have revealed that an increase in Bacteroidetes abundance is independently associated with MASH, whereas an increase in Ruminococcus abundance is independently associated with fibrosis (12). Compared with healthy individuals, patients with MASLD exhibit a significant reduction in intestinal flora diversity, significant changes in intestinal flora composition, a remarkable increase in the abundance of gram-negative bacteria, and a remarkable reduction in the abundance of Firmicutes (13, 14). A significant correlation between liver fibrosis and a high abundance of Bacteroides and *Escherichia coli* was observed in the metagenomic sequencing data (15). Additionally, an increased abundance of Escherichia, Shigella, and Enterobacteriaceae is closely related to advanced fibrosis (16). The proportion of Bacteroidetes in patients with MASH is lower than that in healthy controls and is unrelated to diet or body mass index (17). Studies have also indicated no changes in the levels of Bacteroidetes between those with MASH and healthy controls (14). Various factors, such as geographical location, diet, age, and study population, may cause these inconsistent results. Therefore, more studies are needed to elucidate the exact mechanism of the interaction between gut microbiota and liver inflammation.

The intestinal flora is easily affected by external factors, such as dietary habits and lifestyle. Long-term high-sugar and high-fat diets can lead to an imbalance in the intestinal flora ecology, damaging barrier function and disrupting immune homeostasis (18). Many bacteria, along with their metabolites and cytokines, enter the liver via the portal vein, exceeding the processing capacity of the mononuclear macrophage system (19, 20), triggering a cytokine cascade reaction, inducing excessive activation of immune cells, releasing large amounts of inflammatory mediators, exacerbating damage, inflammation, and fibrosis, and accelerating the development of MASLD (21, 22). Therefore, the gut microbiota is a key factor in MASLD pathogenesis.

## 3 Metabolites of gut microbiota and MASLD

### 3.1 Bile acids

In liver cells, bile acids are synthesized from cholesterol through a series of enzymatic reactions, secreted into the bile, and released into the intestine to promote the emulsification and absorption of dietary fat, cholesterol, and fat-soluble vitamins. The gut microbiota regulates bile acid metabolism through the bile acid receptors farnesol X receptor (FXR) and G protein-coupled bile acid receptor 5 (TGR5). This regulation involves gene expression related to bile acid synthesis, metabolism, and reabsorption, and it plays a critical role in maintaining liver glucose, lipid, and energy metabolism (23).

FXR-mediated signaling has favorable effects on carbohydrate metabolism and hepatic lipid. FXR is activated by primary bile acids. After activation, it stimulates the peroxisome proliferator-activated receptor α (PPARα) expression, induces the expression and secretion of fibroblast growth factor 21 (FGF21), and activates the mammalian target of rapamycin. FXR enhances glucose uptake in adipocytes and stimulates fatty-acid oxidation in differentiated adipocytes by modulating the activity and stability of PPARγ, the main transcriptional regulator of adipogenesis (24, 25). In mice fed with a high-fat diet (HFD) to model MASLD, FXR reduced hepatic lipogenesis by regulating the intestinal antagonism of gut microbiota (26). Activating FXR receptors can change the gut microbiota, especially gram-positive bacteria, e.g., *Streptococcus thermophilus*, *Lactobacillus lactis*, and *Lactobacillus casei* (27). The FXR agonist obeticholic acid prevents intestinal barrier damage and improves MASH (28, 29). A phase III clinical trial of MASH combined with fibrosis revealed that obeticholic acid significantly improved the degree of fibrosis (30). These studies suggest that FXR receptor agonists, as well as tissue-selective FXR activation, could be promising targets for the prevention and treatment of metabolic syndromes, including fatty liver disease and MASH.

TGR5 is activated primarily by secondary bile acids. Kupffer and endothelial cells express TGR5 in the liver tissue and regulates liver inflammation and glucose metabolism. TGR5 reduces inflammatory responses by inhibiting nuclear factor kappa-B (NF-κB) activity and cytokine production in macrophages (31). The intestinal microbiota affects the homeostasis of the bile acid pool by metabolizing the main bile acids into secondary bile acids, which regulate lipid and energy metabolic pathways in MASLD. In mice fed a Western diet, the selective TGR5 agonist RDX8940 improved insulin sensitivity and liver steatosis (32). Moreover, BAR502 is a non-steroidal dual FXR and TGR5 agonist that stimulates white adipose tissue browning and reverses liver steatosis inducted in HFD-fed mice (33).

Recent studies have reported bile acids as key nutrient sensors and metabolic integrators that play important roles in maintaining metabolic homeostasis. The intestinal microbiota can regulate the incidence and development of MASLD via BA metabolism and FXR/TGR5 signal transduction pathways, providing basic evidence for intestinal flora-targeted treatment of MASLD.

### 3.2 Short-chain fatty acids

Short-chain fatty acids (SCFAs) are saturated fatty acids composed of five or fewer carbon atoms. SCFAs are absorbed and delivered to the liver via the portal vein and affect liver lipid metabolism through a protein kinase (adenosine monophosphate kinase, AMPK)-dependent mechanism activated by adenosine monophosphate, participating in the occurrence and development of MASLD (34). Additionally, SCFAs exert immunomodulatory effects on Treg cell differentiation by dysregulating histone deacetylase and the G protein-coupled receptor 43 (GPR43) pathway. It reduces the migration and proliferation of various immune cells, such as macrophages, neutrophils, T lymphocytes, and monocytes. It also reduces the expression of pro-inflammatory cytokines, upregulates the anti-inflammatory cytokine prostaglandin E2, and exerts anti-inflammatory effects (35). In the liver, SCFAs also promote energy consumption and fat oxidation, affecting the host energy supply and metabolic homeostasis (36). Dietary supplementation with SCFAs prevents and reverses metabolic abnormalities induced by HFD-fed mice (37).

SCFAs are derived from the fermentation of dietary fiber by intestinal bacteria, among which butyric acid and acetic acid, have the highest contents in the intestine (38). Acetate and propionate are produced by Bacteroidetes in the intestine and play key roles in hepatic lipogenesis and gluconeogenesis. Propionate supplements can significantly reduce body weight and intrahepatic lipid content, have beneficial effects on β-cell function in the body, and stimulate human colon cells to release polypeptide-YY and glucagon-like peptide 1 (GLP-1) (39). Acetate affects GPR, GPR43, and GPR41. These receptors are distributed in intestinal endocrine L-cells, white adipocytes, skeletal muscle, liver, and pancreatic β cells. L-cells release GLP-1, which acts directly on liver cells, activating genes related to fatty acid β-oxidation, thereby contributing to MASLD development (40, 41).

Butyric acid is primarily produced by Firmicutes. Butyrate can activate AMPK and improve intestinal flora imbalance to alleviate steatohepatitis induced by a high-fat diet (42, 43). Animal experiments (44) have demonstrated that butyrate-producing probiotics correct enterohepatic immune disorders and MASH caused by a HFD, and this effect is mediated by SCFAs. Butyrate supplementation can alleviate high-fat diet-induced MASH, and its potential mechanism involves improving intestinal flora imbalance and gastrointestinal barrier function, thereby hindering the transport of intestinal-derived endotoxins to the liver (42). Oral administration of sodium butyrate inhibits liver inflammation in mice, thereby preventing MASH development (45).

Increasing the SCFAs derived from dietary fiber fermentation is an important strategy for preventing and alleviating MASLD. However, whether reduced SCFA production due to gut dysbiosis is a major factor exacerbating hepatic metabolic disorders remains unclear. Moreover, supplementation with SCFAs alone does not always alleviate fat metabolism disorders, which may be related to individual differences in the intervention time, SCFA type, or health status. Research on the regulatory effects of SCFAs on the host has improved the understanding of the relationship between MASLD and SCFA; however, further research is still needed.

### 3.3 Lipopolysaccharides

Lipopolysaccharides (LPS), an endotoxin, is the main part of the outer membrane of gram-negative bacteria (46). Overgrowth of gram-negative intestinal bacteria is associated with increased intestinal permeability (47). Gut microbiota disorders destroy the integrity of the intestine and impair intestinal barrier function, allowing LPS produced by intestinal bacteria to enter the portal vein via the blood flow dynamics, thus promoting the liver’s inflammatory response. Increased LPS damages the intestinal barrier through toll-like receptor (TLR)-dependent upregulation of myosin light chain kinase and activation of interleukin-1 receptor-associated kinase function, causing increased intestinal permeability. LPS and its downstream pathways substantially affect liver inflammation in MASLD (48, 49). TLR4, widely expressed in hepatocytes, is a pattern-recognition receptor for LPS and various free fatty acids. LPS reaches the liver via the portal vein and induces TLR4 activation. Once TLR4 is triggered, the essential adapter protein myeloid differentiation primary response 88 (MyD88) is simultaneously activated, leading to the activation of NF-κB, which triggers inflammation and promotes the release of inflammatory factors (50–52). When liver Kupffer cells are exposed to bacterial LPS, they release pro-inflammatory cytokines and chemokines by activating TLR4, MyD88, and NF-κB pathways, stimulating stellate cells, and promoting hepatic stellate cells and fibrosis formation (53, 54). Therefore, the activation of the TLR4-mediated NF-κB inflammatory pathway induced by gut-derived bacterial LPS may be key to MASH development.

The intestinal permeability of patients with MASLD is double that of normal individuals, and this abnormality is related to excess bacteria build up and the destruction of tight junction integrity in the small intestine (55). LPS activity in serum is elevated in patients with MASLD, with increases of 38–40% compared with those in patients with metabolic disorders without MASLD (48, 56). LPS from biopsy-proven human MASLD showed increased localization in hepatocytes, considerably associated with inflammation of the liver via the TLR4 pathway (48). Reducing the plasma LPS levels can improve hepatic steatosis, suggesting that chronic low-grade inflammation induced by LPS is an important factor in MASLD progression.

### 3.4 Endogenous ethanol from gut microbiota sources

In a healthy state, the microbiota continuously produces ethanol in the intestine, which is metabolized by alcohol dehydrogenase enzymes in liver. As a metabolite of gut microbiota, endogenous ethanol blocks the tricarboxylic acid cycle and increases acetate levels, promoting the accumulation of triglycerides in hepatocytes (57). Acetaldehyde, the product of ethanol metabolism, is involved in destabilizing intestinal tight-junction proteins and is related to the downregulation of antimicrobial peptide expression in the intestine (58). It also increases intestinal barrier permeability and LPS levels, activates TLRs and inflammasomes, and aggravates liver damage (59). Additionally, ethanol can directly damage the liver after absorption. Ethanol causes P450 2E1 mRNA and protein over-expression, leading to free radical formation, mitochondrial dysfunction, and liver damage (57).

The blood ethanol level of children with MASLD is remarkably higher than that of healthy children and is positively correlated with the levels of leptin, and triglyceride in the blood (60). In MASH patients who do not consume alcohol, variations in the composition of the intestinal flora causing dysbiosis increase the blood levels of insulin, leptin, and triglyceride. This finding suggests dysbiosis may lead to endogenous ethanol production via intestinal microbial fermentation (61). Further analysis revealed that the gut microbiota of MASH patients contained the Escherichia genus, Proteobacteria, and Enterobacteriaceae, which have ethanol-producing functions and was significantly higher in patients with obesity than in healthy individuals. Preclinical and clinical studies have identified *E. coli*, Enterobacteriaceae, and *Klebsiella pneumoniae* as ethanol-producing bacteria that are abundant in mice and patients with MASLD (62). For example, studies using a highly alcohol-producing *K. pneumoniae* strain W14 demonstrated that its mutant W14-Δadh can induce steatosis in HepG2 hepatocytes, reduce adenosine triphosphate content, increase mitochondrial reactive oxygen species accumulation, and cause DNA damage. Additionally, mouse hepatocytes have been observed in animal experiments (liver and mitochondrial damage) (57). Transplanting fecal microbiota containing a strain of *K. pneumoniae* (HiAlc Kpn) isolated from individuals with MASLD into mice and selectively eliminating the HiAlc Kpn strain before fecal microbiota transplantation (FMT) can prevent the development of MASLD in recipient mice, demonstrating that changes in the gut microbiota lead to excess endogenous alcohol production (63).

MASLD and alcohol-associated liver injury share common histological features and similar pathogenic pathways. The regulation of the gut bacteria produces various metabolites, eventually leading to MASLD development. However, the role of endogenous ethanol requires further in-depth research using larger clinical samples.

### 3.5 Choline

Choline is an essential phospholipid for the human body. It is mainly absorbed through the diet and synthesized by the liver. It plays an important role in hepatic lipid transport (64). Choline deficiency inhibits the synthesis and secretion of very low-density lipoproteins, resulting in triglyceride accumulation and hepatic steatosis (65, 66). Choline can be metabolized by intestinal flora, such as *Proteus penneri*, *E. coli*, and *Proteus mirabilis*, which cleave the carbon-nitrogen bond and convert choline into trimethylamine (TMA). TMA is then oxidized by liver monooxygenase to form trimethylamine N-oxide (TMAO), which reduces phosphatidylcholine levels in the blood, reduces the host’s choline bioavailability, and exposes the host to inflammatory and toxic metabolites. This process mimics a choline-deficient state and leads to metabolic disorders (67). According to a previous report (68), TMAO serum levels are elevated in patients with MASLD. TMAO regulates glucose metabolism and induces insulin resistance by increasing serum levels of the chemokine C–C motif ligand 2, causing adipose tissue inflammation and abnormal blood sugar levels. Furthermore, a clinical study (69) has revealed that higher serum TMAO levels positively correlate with MASLD severity.

After ingesting foods containing choline, microorganisms in the intestine, including gram-positive and gram-negative bacteria, synthesize TMA. Therefore, TMA production is affected by individual’s microbiota composition. Less than 1% of the microbes in the intestines carry the genes necessary for TMA production (70); however, even very low-density of these microorganisms are sufficient to produce TMA (71). Increased TMA and TMAO levels are correlated with higher activity of bacterial members of the phyla Firmicutes and Proteobacteria. Additionally, increased levels of TMA and TMAO are linked to increased Firmicutes/Bacteroidetes ratios (72). A human experiment controlling choline intake showed that the composition of the gut microbiota changes with alterations in dietary choline levels, among which γ-Proteobacteria and Erysipelothrix are related to the changes in liver fat during choline consumption (73). A choline-deficient diet is connected with MASH and may lead to obesity (74), and mouse experiments have revealed that changes in the composition of the bacterial community may be associated with choline depletion and an increase in toxic methylamine (75). *Enterobacter aerogenes* is another bacterial strain that effectively reduces plasma and cecal TMAO levels by altering the ratio of commensal to pathogenic bacteria in in choline diet-fed mice (76). Research on the intestinal microbiome may improve our understanding of nutritional metabolism and the impact of diet on health. Nutrition-based personalized approaches that target changes in gut microbial structure and function can help better understand the interaction between intestinal flora and metabolic diseases.

## 4 Potential preventive and therapeutic effects of intestinal flora on MASLD

As no specific method has been established for treating MASLD, lifestyle intervention is the most basic method, especially diet and exercise (77). Probiotics, prebiotics, synbiotics, postbiotics, FMT, next-generation probiotics (NGPs), and water consumption can modulate the intestinal microbiome and its effect on the gut-liver axis in patients with MASLD. Extensive research has been conducted in animal models and clinical trials, achieving effective results and good prospects. These approaches are expected to become new methods for preventing and treating MASLD.

### 4.1 Probiotics

Live microorganisms that provide health benefits to the host are probiotics (78). They can act on different target organs by generating antimicrobial peptides, decreasing intestinal permeability, and inhibiting the translocation of bacterial products (79). They affect intestinal mucosal immune function in patients or models with fatty liver disease. There are many types of probiotics that are frequently used in the medical field, among which *Lactobacillus* and *Bifidobacterium* are the most commonly used. Lactobacilli and bifidobacteria are associated with β-glucuronidase inhibition (80), and bifidobacteria prevent pro-inflammatory cytokine secretion and intestinal barrier dysfunction (81).

Animal studies have revealed that supplementation with probiotic preparations can improve intestinal epithelial permeability, maintain tight junction proteins, reduce inflammation, and reduce liver triglyceride concentrations (82). Lactobacilli can activate the AMPK pathway to phosphorylate acetyl-CoA carboxylase (ACC), block the sterol regulatory element binding protein 1 (SREBP-1)/fatty acid synthase (FAS) signaling pathway, and inhibit fat metabolism. It can also positively affect liver damage mediated by c-Jun N-terminal kinase and NF-κB (82, 83). *Lactobacillus sakei* MJM60958 significantly reduces the expression of genes and proteins involved in fat accumulation, such as ACC, SREBP-1, and FAS, and increases the expression of proteins related to lipid oxidation, such as carnitine palmitoyltransferase 1a and PPARα (84). *Lactobacillus plantarum* ZJUIDS14 can increase the expression of fatty acid transporter 2, fatty acid transporter 5, and SREBP-1C and promote fatty acids biosynthesis and triglyceride accumulation (85). *Bifidobacterium* L66-5, FS31-12, M13-4, and L75-4 have been shown to decrease the serum and liver triglyceride levels; however, only *Bifidobacterium* FS31-12 and L66-5 substantially reduced their levels in the liver.

In a clinical study, Alisi et al. (86) discovered that supplementing with VSL #3 (containing *Lactobacillus paracasei*, *Bifidobacterium longum*, *Bifidobacterium breve*, and *Lactobacillus acidophilus*), bifidobacteria, and *Streptococcus salivarius* for 4 months improved liver function and increased GLP/active GLP levels in obese children with MASLD. It should be noted that the post-2016 VSL#3 probiotic formulation differs from the De Simone Formulation, which was commercially available under the trademark VSL#3^®^ only until 2016 (87). Sepideh et al. (88) reported that supplementation with multi-strain probiotics can contribute to improvement of insulin sensitivity and liver inflammation in MASLD. Additionally, combining probiotics and drugs, such as statins and metformin, can improve liver inflammation and lower cholesterol levels better than using them alone (89). These studies suggest that probiotics alone or combined with other drugs have potential for clinical application in MASLD treatment. Consumption of *Bacillus bulgaricus* and *S. thermophilus* decreases the abundance of *Firmicutes*, *Clostridium*, and *Erysipelotoxalis* genera, whereas it increases the relative abundance of *Selenomonas* (90). However, in a clinical study conducted in Malaysia, patients with MASLD were supplemented with multi-strain probiotics (BCMC strain) for 6 months. The use of probiotics did not have a major impact on patients with MASLD.

However, at the microenvironmental level, probiotics appear to stabilize mucosal immune function, improve intestinal mucosal morphology, and protect patients from increased gut permeability (91). Different probiotic products and dosages have different effects on intestinal microbial composition. Differences in local intestinal microbiota also affected the results of the study. Therefore, a larger sample size is required for similar studies.

### 4.2 Prebiotics

Prebiotics are edible food ingredients composed of polysaccharides and oligosaccharides that help grow beneficial bacteria and regulate changes in the intestinal microbial communities. Lactulose, inulin derivatives, fibers, and lactooligosaccharides are the currently available prebiotics (92). Prebiotics (inulin and fructooligosaccharides) are considered safe in the United States and most European countries (93). A review has investigated the efficacy of prebiotics in MASLD treatment (94). Dietary oligofructose stimulates the reproduction of good bacteria (*Bifidobacterium* genus) and stimulates fatty acid oxidation through PPARα to reduce liver triglyceride accumulation. It also reduces cholesterol accumulation by hindering SREBP-2-dependent cholesterol biosynthetic pathways (95). Gellan gum, an anionic polysaccharide with prebiotic activity, is used as additives in foods. Thus, gellan gum may support liver health by regulating intestinal homeostasis (96). Additionally, pectin modulates the intestinal microbiota and protects the liver against metabolic damage induced by alcohol and fatty diets (97). In a clinical study, patients with MASLD who were treated with prebiotics exhibited significant reductions in hepatic steatosis and MASLD activity scores and increased bifidobacterial counts compared with the placebo group (98). In a study on adult MASLD, participants received either 20 g of inulin control or inulin propionate daily for 42 days. The study revealed a significant increase in intracellular lipids in liver cells in the inulin control group, whereas no significant changes were observed in the inulin propionate group, indicating that although supplementation with inulin propionate did not reduce liver fat, it significantly mitigated the liver fat increase caused by inulin supplementation (99). Prebiotic intake benefits the health of humans by modulating the gut microbiota and is safe and well tolerated. However, some researchers have reported concerns regarding prebiotic supplements, as high doses of these compounds (i.e., 30 g/day) may cause negative gastrointestinal reactions, mainly flatulence (100).

Animal studies have demonstrated that prebiotic supplementation can reduce fatty acid synthesis pathways, thereby reducing hepatic triglyceride accumulation, possibly because of decreased gene expression of enzymes that regulate lipogenesis (ACC and FAS) (101). As a prebiotic, L-arabinose alters gut microbiota diversity, thereby improving body fat percentage, blood lipid levels, fasting blood glucose, and liver damage in animals with metabolic syndrome models (102, 103). Combination treatment with isomaltooligosaccharides and lycopene prevented excessive weight gain, enhanced fat mobilization from adipose tissue, improved insulin resistance, and reduced metabolic endotoxemia in mice with HFD-induced MASLD, suggesting that the combined use of antioxidants and prebiotics is more beneficial in MASLD treatment. Moreover, in obese Zucker rats, a diet rich in oligofructose and raspberry polyphenol extract has adequate health-promoting potential to regulate oxidative stress and inflammation associated with MASLD development. Prebiotics can selectively stimulate the proliferation and activity of intestinal microorganisms and may be an alternative direction for human health in the future, but overall, compared with probiotics, research on the application of prebiotics for MASLD is limited (104).

In addition, *Hericium erinaceus* is a fungus with prebiotic activity. In recent years, multiple studies have indicated that *H. erinaceus* may be a potential manipulator of gut microbiota, providing essential nutrients and regulating the ecological balance of human gut microbiota (105, 106). A pilot study showed that supplementing *H. erinaceus* for 7 days increased alpha diversity within the gut microbiota, upregulated the relative abundance of some SCFA bacteria (*Kineothrix alysoides*, *Gemmiger formicilis*, *Fusicatenibacter saccharivoras*, *Eubacterium rectale*, and *Faecalibacterium prausnitzii*), and downregulated some pathogenic bacteria (*S. thermophilus*, *Bacteroides caccae*, and *Romboutsia timonensis*) (107). During the digestion and fermentation process under simulated gastrointestinal conditions *in vitro*, bioactive water-soluble polysaccharide and alkaline soluble polysaccharide from *H. erinaceus* were found to increase the relative abundance of dominant butyric acid-producing genera, regulate microbial-community structure, increase gas production and SCFA production in the fermentation broth, and lower the pH value of the fermentation broth (108). As a supplementary food, *H. erinaceus* can regulate the composition of gut microbiota and have beneficial effects on health. However, regarding the currently limited research on *H. erinaceus* intervention in MASLD, further clinical and experimental studies are still needed.

### 4.3 Synbiotics

Synbiotics are combinations of prebiotics and probiotics (80, 109) that selectively stimulate the growth of certain beneficial bacteria and initiate their metabolism, resulting in positive effects (110). Many studies have reported the benefits of synbiotics in treating MASLD, such as improved liver steatosis levels, reduced liver inflammation, and improved alanine aminotransferase (ALT) parameters (111, 112). In a randomized placebo-controlled trial of 50 patients, researchers used a synbiotic composed of multiple *Lactobacillus* and *Bifidobacterium* strains and observed greater reductions in liver cirrhosis (111).

Some researchers have combined synbiotics with drugs to treat MASLD and achieved good results. Synbiotics have a synergistic effect with the Chinese herbal medicine *Sonchus brachyotus* DC extract, and the combination of the extract and synbiotics proved substantially more effective in treating MASLD than either component alone (113). Combining proanthocyanidins with probiotics to develop synbiotics can slow down the progression of steatosis to MASH by reducing liver oxidative stress, liver damage, and inflammation caused by gut floral dysbiosis. Proanthocyanidin synbiotics are more effective at reducing the possibility of MASLD than proanthocyanidins or probiotics alone (99). Synbiotics also improve blood glucose and insulin levels (114).

Several researchers have conducted retrospective analyses of the use of synbiotics to treat MASLD. A 2013 meta-analysi**s** demonstrated the positive effects of probiotics in lowering ALT and total cholesterol levels (115). A recent meta-analysis involving in 782 patients with MASLD revealed that supplementation with probiotics and synbiotics was beneficial for liver steatosis, blood lipids, and liver fibrosis; however, supplementation was unable to improve body mass index, fasting blood glucose, and waist circumference (116). Another systematic review study obtained similar results, revealing that supplementation with synbiotics could reduce body weight, fasting blood glucose, insulin, total cholesterol, triglycerides, high-sensitivity C-reactive protein, ALT, aspartate aminotransferase levels, and low-density lipoprotein cholesterol in patients with MASLD. However, compared with the placebo group, synbiotics showed no beneficial effects on waist circumference, body mass index, homeostasis model assessment of insulin resistance, and high-density lipoprotein cholesterol levels (117). Furthermore, a recent study in the UK involving 104 patients observed no significant difference in liver steatosis between synbiotic preparations and placebo; taking a synbiotic combination for 1 year changed the fecal microbiome; however, it did not decrease the liver fat content or liver fibrosis markers (118).

Synbiotics exert a synergistic effect between probiotics and prebiotics, providing the host with greater advantages. This synergistic effect selectively enhances the growth and activation of health-promoting bacteria in the intestine and cultivates a more favorable microbial environment, thereby improving immunity and relieving symptoms including bloating and abdominal pain caused by lactose intolerance. It is expected to become an important method for preventing and treating metabolism-related diseases.

### 4.4 Postbiotics

Compared with traditional functional foods, such as probiotics and prebiotics, postbiotics have the advantages of including a single ingredient and having high physiological activity, long shelf life, and easy absorption properties. So far, data on human postbiotic research has only involved butyrate salts. Butyric acid is a key metabolite produced by the microbiome in the large intestine through the breakdown of indigestible carbohydrates. Butyrate can positively regulate the expression of claudin-1, ZO-1, and occludin in Cdx2-IEC and Caco-2 cells, leading to increased transepithelial resistance and enhanced the mucus layer involved in the formation of the intestinal barrier (119, 120). A double-blind clinical trial showed that a dietary supplement based on butyrate (calcium butyrate 500 mg/tablet) can improve some MASLD-related parameters affected by hepatic steatosis and metabolic syndrome (121). In animal experimental research, butyrate enhances liver GLP-1 sensitivity by increasing GLP-1 receptor expression, thereby alleviating liver steatosis (122).

In addition to butyrate derived from gut microbiota, other postbiotics derived from traditional probiotics represented by *Lactobacillus* and next-generation probiotics (NGPs) represented by *Akkermansia* have been studied. The oral administration of postbiotics prepared from *L. paracasei* effectively prevent MASLD in mice (123). Bacterial sequencing showed that postbiotics regulated the gut microbiota, increased the relative abundance of *Akkermansia*, and decreased the relative abundance of *Lachnospiraceae*, *Ruminiclostridium*, and *Bilophila*. The postbiotics derived from the mucinous protein of *Akkermansia* play a crucial role in regulating metabolic functions to prevent obesity (124). Thus, postbiotics can alleviate diseases and protect host health. However, the mechanism by which postbiotics prevent MASLD needs further investigation.

### 4.5 FMT

FMT is a new method for restoring and reconstructing the balance and diversity of intestinal microecology. It is used to transplant functional intestinal flora from healthy human donor stool into the intestines of patients. Various animal-based studies have revealed that FMT can competently improve the symptoms of MASLD by changing the intestinal flora imbalance (125–127). FMT reduced the inflammation of liver in HFD-induced mouse MASH model by improving intrahepatic fat accumulation and serum pro-inflammatory cytokine levels. Le Roy et al. (128) discovered that FMT in different mouse models caused germ-free mice to exhibit different lipogenesis and steatosis phenotypes.

The use of FMT in treating patients with MASLD is gaining attention. FMT can reduce excess fat storage in the liver by improving the intestinal flora imbalance, thus alleviating fatty liver disease. Some patients with chronic diarrhea and constipation symptoms were relieved through FMT, and the effect of FMT on gut microbiome reconstruction in lean patients with MASLD was better than that in obese patients with MASLD (129, 130). FMT from a healthy donor may affect levels of genes engaged in liver inflammation and lipid metabolism (131). FMT replenishes the balance of the intestinal microbial environment and rebuilds bacterial colonization, thereby restoring microbial richness and preventing excessive influx of bacterial products into the liver. Restoration of the intestinal barrier function can ameliorate lipid metabolism, reduce insulin resistance, and inhibit inflammatory responses, thereby alleviating MASLD (130, 132). FMT is safe for long-term use. However, there are reports of adverse events including death in patients undergoing FMT. A systematic review of FMT showed that the proportion of adverse events was similar between immunocompromised and immunocompromised patients (133). Four patients were reported to have gram-negative bacteremia after undergoing FMT. Furthermore, a clinical study described two patients in whom extended-spectrum beta-lactamase-producing *E. coli* bacteremia occurred after having undergone FMT (134). Genomic sequencing indicated that both cases were related to the same fecal donor. Therefore, it is necessary to strengthen donor screening and limit microbial transmission that may lead to adverse infection events. In the future, FMT should be personalized for different patients and situations based on different hosts and diseases.

### 4.6 NGPs

NGPs include living microorganisms that are beneficial to the health of the host (135). *Akkermansia* and *Christensenella minuta* are among the most widely used and researched NGPs. *Akkermansia muciniphila* is involved in intestinal mucosa regeneration and intestinal-barrier integrity regulation, which alters the composition of the gut microbiota, reduces the intestinal infiltration of inflammatory mediators and harmful substances, and thus alleviates liver damage (136). In human studies, evidence shows that *A. muciniphila* abundance is negatively related to the risk factors of MASLD, such as being overweight, obesity, and untreated type 2 diabetes (137). *A. muciniphila* also regulates inflammation by modulating TLR2-activated gamma delta T17 cells and may affect the transition of macrophages from a pro-inflammatory state to an anti-inflammatory state (138). These changes help reduce inflammation and prevent the progression of MASH. Furthermore, *Christensenella minuta* has potential therapeutic effects in MASLD (139), and its abundance in obese individuals was lower than that in lean individuals (140).

A study based on the effects of traditional probiotics and NGPs on MASLD/MASH showed that traditional probiotics mainly reduce liver fat deposition and inflammation by improving gut microbiota composition and enhancing intestinal barrier function (141). In contrast, NGPs exhibit more significant therapeutic potential. NGPs are not limited to regulating gut microbiota, liver oxidative stress, and inflammatory response (142, 143). They can also produce bioactive compounds such as SCFAs (144) and regulate bile acid metabolism, which in turn activates nuclear receptors and signaling pathways in the liver (such as FXR and TGR5), indirectly regulates signaling pathways related to oxidative stress in liver cells, and helps improve liver inflammation (145). In recent years, NGPs have shown great therapeutic potential in the treatment of MASLD/MASH (146). With the advancement of technology and the emergence of microbiome research, the use of NGPs is a new potential strategy for managing MASLD/MASH.

### 4.7 Water consumption

Natural mineral water is rich in minerals and elements, such as calcium, carbonate metabolites, sodium chlorite, sulfates, and iron, making it a valuable means of consuming essential elements in the diet (147). The use of natural mineral water for therapeutic purposes has been proposed as a useful supplement for managing various gastrointestinal and hepatobiliary diseases (148). Research has confirmed that bicarbonate contributes to the intestinal barrier structure, and the use of bicarbonate-rich water has a positive impact on intestinal histopathology (149). Sulfate saline administration can also promote the production of hydrogen sulfide by sulfate reducing bacteria (such as *E. coli*) in the intestinal lumen (150). In a prospective longitudinal intervention study, bicarbonate-sulfate-calcium-magnesium water was shown to have a positive effect on indirect markers of gut-liver axis activation and alterations in gut microbiota in patients with MASLD (151). A histopathological study investigated the effects of calcium-sulphate-bicarbonate water treatment on MASLD; mineral water treatment was associated with improved intestinal mucosal histopathology and increased positive levels of closing proteins, indicating that the LPS and TLR4 pathways mediated gut liver axis and regulated inflammatory damage (152). As a microbiota-modifying strategy in patients with MASLD, mineral water has regulatory activity on the gut-liver axis and potential beneficial effects.

## 5 Discussion

Changes in gut microbiota affect the development and progression of MASLD. Clinical trials and animal models provide ample evidence elucidating the role of gut microbiota in MASLD pathogenesis. Abnormal alterations in gut microbiota structure and metabolic products, as well as changes in intestinal mucosal permeability, increase the likelihood of intestinal pathogens entering the liver through the hepatointestinal axis. This leads to metabolic disorders and further exacerbates liver cell damage caused by inflammatory reactions. Although specific changes in gut microbiota observed in many studies are not unique to MASLD, it is evident that gut microbiota holds potential as a target for clinical treatment of MASLD. Maintaining a balanced gut microbiota can help alleviate liver inflammation and delay the development of MASLD. Future research should focus on exploring the relationship between gut microbiota composition changes and MASLD, identifying microbial species associated with MASLD, clarifying the characteristics of the gut microbiota in patients with MASLD, and elucidating the mechanisms by which gut microbiota abnormalities can affect MASLD. This will pave the way for the development of specific drugs and effective treatment methods for MASLD.

Studies on intestinal flora have been conducted for decades, and those in recent years have greatly enriched our understanding of intestinal flora, especially the relationship between gut microbiota and liver diseases. Although many studies have revealed that gut microbiota can affect the incidence of MASLD through the gut-liver axis, the mechanism by which intestinal flora imbalance affects MASLD remains unclear. The composition of gut microbiota in patients with MASLD shows changes; however, these changes have not been fully characterized. The connection between intestinal flora and the pathogenesis of MASLD needs further study. Moreover, the relationship between the gut and the liver is bidirectional, and changes in the composition of gut microbiota may not necessarily be the cause of changes observed in the liver. Furthermore, liver disease itself can affect the composition of gut microbiota; therefore, results of research on the association between gut microbiota and liver disease should be interpreted with caution. Besides, gut microbiota can vary depending on the demographics and disease stage of MASLD, challenging conclusive claims regarding the richness and certain bacterial species in the gut microbiota of those with MASLD. It is necessary to accurately characterize the extensive microbial changes based on the pathological characteristics of each stage of MASLD and to conduct sufficient large-scale intervention studies in target patients with reproducibility to reveal the correlations between microbial community intervention and MASLD management.

In terms of prevention and treatment, targeted intestinal flora, such as probiotics, prebiotics, synbiotics, postbiotics, FMT, NGPs, and water consumption, have achieved positive therapeutic effects in animal studies, and related clinical studies have gradually received attention. However, some problems must be resolved before these can be used as routine treatment options. Because of the risk of disease transmission between donors and recipients, standardization of donor screening, patient acceptance, adverse outcomes, and uncertain effects on recipient immunity, an FMT registry should be established to collect long-term data, follow-up results, and adverse event monitoring. Furthermore, many studies have focused on animal experiments, and clinical trials are lacking. Intestinal flora differs among regions, diets, and patients. The clinical efficacy of gut microbiota-targeted therapy for MASLD must be demonstrated in randomized controlled trials to prove the feasibility of probiotics, prebiotics, synbiotics, postbiotics, FMT, NGPs, and water consumption therapy.

Additionally, as a novel potential therapy, postbiotics have advantages, such as including a single ingredient and having high physiological activity and long shelf life, compared with traditional functional foods. Furthermore, the use of post-biological agents carries less risk and is more suitable for geographical areas where reliable cold chain or high temperatures pose storage challenges for live microorganisms. Therefore, they have more potential applications. Many clinical trials have demonstrated the positive effects of inanimate microorganisms on hosts; however, there remains a notable gap in clinical research on microbial metabolites and cellular components. In the future, research should focus on animal models or clinical trials, more studies should be conducted to understand the host’s mechanism of action, and the safe dosage range for use should be determined to enhance safety assessment.

In summary, owing to their strong biological activity, microorganisms have a wide range of potential applications in the development of functional foods and drugs. However, more research is needed to determine their true efficacy. Further exploration of the prevention and treatment of liver diseases such as MASLD by regulating the intestinal flora is expected to become an area of focus for future research.
